# Bevacizumab Efficacy in Metastatic Colorectal Cancer is Dependent on Primary Tumor Resection

**DOI:** 10.1245/s10434-013-3463-y

**Published:** 2014-01-14

**Authors:** Francois Ghiringhelli, Damien Bichard, Samuel Limat, Veronique Lorgis, Julie Vincent, Christophe Borg, Julie Berthou, David Orry, Pablo Ortega-Deballon, Zaher Lakkis, Olivier Facy, Bruno Heyd, Patrick Rat, Virginie Nerich, Sylvain Ladoire

**Affiliations:** 1 Department of Medical Oncology, Centre Georges-François Leclerc, INSERM Avenir 866, Dijon, France; 2 Institut National de la Santé et de la Recherche Médicale (INSERM), UMR-866, University of Burgundy, Dijon, France; 3 Institut National de la Santé et de la Recherche Médicale (INSERM), UMR-1098, Besançon, France; 4 Department of Pharmacy, University Hospital of Besançon, Besançon, France; 5 Institut National de la Santé et de la Recherche Médicale (INSERM), UMR 1098, University of Franche Comté, Besançon, France; 6 Department of Medical Oncology, University Hospital of Besançon, Besançon, France; 7 Department of Oncologic Surgery, Centre Georges-François Leclerc, Dijon, France; 8 Department of Digestive and Oncologic Surgery, University Hospital of Dijon, Dijon, France; 9 Department of Digestive and Oncologic Surgery, University Hospital of Besançon, Besançon, France

## Abstract

**Purpose:**

Bevacizumab plus fluoropyrimidine-based chemotherapy is standard treatment for first-line and second-line metastatic colorectal cancer (mCRC). However, to date, there is no current biomarker predictive for the benefit of bevacizumab use for these patients. Preclinical data suggest that the presence of the primary tumor could be involved in less efficient antitumor activity of antiangiogenic agents, but no clinical data currently support this hypothesis.

**Methods:**

We performed a retrospective analysis of factors associated with overall survival (OS) in a study cohort of 409 mCRC patients. Univariate and multivariate Cox proportional hazard regression models were used to assess the influence of primary tumor resection and bevacizumab use on OS. We evaluated associations linking bevacizumab use and OS among patients who previously underwent or did not undergo primary tumor resection. Results were externally validated in a second independent cohort of 328 mCRC patients.

**Results:**

In the study cohort, bevacizumab use and resection of the primary tumor were associated with improved OS. However, subgroup analyses indicate that bevacizumab did not influence survival of patients bearing a primary colorectal tumor (hazard ratio (HR) 0.98, 95 % confidence interval (CI) 0.60–1.61, log-rank test *P* = 0.6). By contrast, the survival benefit of bevacizumab was restricted to patients who previously underwent primary tumor resection (HR 0.71, 95 % CI 0.55–0.92, *P* = 0.009). Similar results were observed in the validation cohort.

**Conclusions:**

Addition of bevacizumab to chemotherapy is associated with improvement of OS only in patients with primary tumor resection. These data support the rationale to validate prospectively the influence of primary tumor resection on bevacizumab antitumor effect in synchronous mCRC.

**Electronic supplementary material:**

The online version of this article (doi:10.1245/s10434-013-3463-y) contains supplementary material, which is available to authorized users.

There are no curative options in patients with unresectable metastases from colorectal cancer (mCRC), but treatment with systemic chemotherapy improves overall survival (OS).^1^ Therapeutic options currently available rely on three cytotoxic chemotherapies, fluoropyrimidine, oxaliplatin, and irinotecan, associated with targeted-therapy anti–epidermal growth factor receptor (EGFR) or anti–vascular endothelial growth factor (VEGF). The 5-year OS in patients who are diagnosed with unresectable distant metastases ranges between 10 and 20 %.^2^–^4^ In contrast, when metastases can be surgically removed, 5-year OS increases to 20–50 %.^5^ Recent clinical studies suggest that survival continues to improve with the routine addition of targeted therapies.^6^–^8^ For instance, phase III trials have shown that adding bevacizumab to first- or second-line chemotherapy may modestly but significantly improve mCRC patient survival. Moreover, the continued use of bevacizumab beyond disease progression leads to a significant improvement in OS and progression-free survival compared with postprogression chemotherapy alone.^6^,^8^–^11^ The association between survival improvement and bevacizumab addition to routine chemotherapy regimens has been also confirmed in large populations of patients with mCRC in prospective cohorts in the context of general oncology practice.^12^,^13^ Consequently, VEGF inhibition has become an attractive therapeutic target in patients with mCRC; however, there is no current predictive biomarker for the efficacy and the clinical benefit of bevacizumab in terms of survival improvement in mCRC patients. This becomes an important goal for clinical trial design and should permit their more rational use in patients with mCRC.

Recent preclinical data have demonstrated that molecular mechanisms involved in neoangiogenesis are different in the primary tumor compared to distant metastases.^14^ Furthermore, in nonresected primary tumor animal models, angiogenesis inhibitors targeting the VEGF pathway demonstrated antitumor effects but concomitantly elicited more invasive or metastatic behavior.^15^

Thus, these data led us to hypothesize that the presence of primary tumor may negatively affect the efficacy of anti-VEGF targeted therapy, leading to lower clinical efficacy. To answer this question, we designed a retrospective clinical study to assess whether the resection of the primary tumor before starting chemotherapy could be associated with patient outcome in mCRC patients receiving chemotherapy with or without bevacizumab.

## Patients and Methods

### Patients

From January 2001 to December 2011, 409 consecutive patients with histologically proven metastatic colorectal adenocarcinoma received first-line chemotherapy treatment at the Georges-François Leclerc Cancer Center (Dijon, France) and were prospectively recorded in an institutional clinical database, which made them eligible for this retrospective study. As first- or second-line treatment, 233 patients were provided chemotherapy plus bevacizumab (bevacizumab group), while 176 patients received chemotherapy alone without bevacizumab (chemotherapy-alone group). Forty-three patients were included in clinical trials for first- or second-line chemotherapy for metastatic disease involving the use of bevacizumab or cetuximab. This mCRC patient cohort constituted the study cohort. The results obtained for this cohort were validated in an independent validation cohort of 328 consecutive mCRC patients who were treated at Besancon University Hospital from 1997 to 2009.

This retrospective study was approved by our Institutional Review Board, and all data were anonymized. Patients received bevacizumab plus fluoropyrimidine-based chemotherapy (chosen at the clinician’s discretion: single-agent fluoropyrimidine or fluoropyrimidine plus oxaliplatin or irinotecan) until disease progression. The bevacizumab dose was 5 mg/kg every 2 weeks (5-fluorouracil-based regimens) or 7.5 mg/kg every 3 weeks (capecitabine-based regimens). Bevacizumab was administered intravenously, initially over 90 min. If the first infusion was well tolerated, the second was delivered over 60 min; if the 60-min infusion was well tolerated, all subsequent infusions were delivered over 30 min.

### Statistical Analysis

All patients were followed up until death or the date of the end of the study (December 31, 2011). The primary end point was OS, which was defined as the interval between date of first dose of chemotherapy and date of death as reported on medical record or December 31, 2011, whichever occurred first. Survivors were censored at last follow-up.

Median follow-up with its 95 % confidence interval (CI) was calculated by the reverse Kaplan-Meier method. The association between the treatment groups (bevacizumab or chemotherapy alone) and other patient and disease characteristics were examined by the Chi-square test and Fisher’s exact probability test, or the Mann–Whitney test if required.

The Kaplan-Meier estimation was used for calculation of survival probabilities and the log-rank test for comparison of survival curves. Cox proportional hazard regression was used to estimate the hazard ratio (HR) and 95 % CI for univariate and multivariate analysis of OS. Classical prognostic factors in mCRC were systematically examined in analyses and included bevacizumab use, anti-EGFR therapy use, number of metastatic sites, age (continuous), sex, serum carcinoembryonic antigen (CEA) level determined before the first injection of chemotherapy (continuous), B-Raf and K-Ras status, World Health Organization (WHO) performance status, presence of synchronous or metachronous disease, complete resection of all metastases, and primary tumor resection before the beginning of chemotherapy for metastatic disease. No information of the measure of the bulk of the disease was collected.

All predictors with *P* values lower than 0.05 in univariate analysis were used in multivariate analysis (except variables with more than 25 % of missing data). Correlations between co-variables were first tested for eligible variables. To prevent collinearity, when two variables were significantly correlated, one variable was retained according to its clinical relevance or to the value of the likelihood ratio (e.g., evolution and primary tumor resection). All analyses were performed by Stata software, version 11 (StataCorp, College Station, TX). *P* values were two-tailed and were considered significant when less than 0.05.

## Results

### Patients From Study Cohort

Between January 2001 and December 2011, 409 mCRC patients were treated in Georges Francois Leclerc Cancer Center. Of these, 233 patients (57 %) received bevacizumab during their chemotherapeutic treatment for metastatic disease. Patient and tumor characteristics are listed in Table 1. There was no significant difference between the bevacizumab and chemotherapy-alone groups for the main clinicobiological characteristics, except for type of metastatic disease (more synchronous metastatic disease in the bevacizumab group: 54 vs. 67.5 %, *P* = 0.004) and except for the proportion of patients who received 3 or more chemotherapeutic lines, which was significantly higher in the bevacizumab group (79 vs. 58.5 %, *P* < 0.0001). Median follow-up at the data cutoff point was 31 months in the bevacizumab group and 33 months in the chemotherapy group.

**Table 1:** Patient and tumor characteristics (*n* = 409)

Characteristic	Variable	Chemotherapy alone (*n* = 175)	Bevacizumab (*n* = 234)	Overall (*n* = 409)	*P* value
Age (year)	Median [min; max]	66.8 [43; 88]	63.5 [24; 90]	65.5 [24; 90]	**0.06**
	Mean (SD)	66.2 (10.3)	64 (11.5)	65 (11)	
Sex	Male	99 (56 %)	120 (51 %)	219 (53.5 %)	**0.33**
	Female	76 (44 %)	114 (49 %)	190 (46.5 %)	
Death		154 (88 %)	158 (67.5 %)	312	<**0.0001**
WHO PS	0	54 (31 %)	83 (35 %)	137 (34 %)	**0.06**
	1	47 (27 %)	58 (25 %)	105 (25 %)	
	2	19 (11 %)	15 (6 %)	34 (8 %)	
	3	7 (4 %)	2 (0.5 %)	9 (2 %)	
	Unknown	48 (27 %)	81 (33.5 %)	129 (31 %)	
B-Raf status	Wild type	13 (7 %)	93 (40 %)	106 (26 %)	**0.9**
	Mutated	1 (1 %)	11 (5 %)	12 (3 %)	
	Unknown	161 (92 %)	130 (55 %)	291 (71 %)	
K-Ras status	Wild type	13 (7 %)	100 (43 %)	113 (27 %)	**0.92**
	Mutated	9 (5 %)	80 (34 %)	89 (22 %)	
	Unknown	153 (88 %)	54 (23 %)	207 (51 %)	
Evolution	Synchronous	94 (54 %)	158 (67.5 %)	252 (62 %)	**0.004**
	Metachronous	81 (46 %)	76 (32.5 %)	157 (38 %)	
Primary tumor resection	Yes	140 (80 %)	193 (82.5 %)	333 (81.5 %)	**0.47**
	No	35 (20 %)	41 (17.5 %)	76 (18.5 %)	
Complete surgery of metastases	No	137 (78 %)	167 (71 %)	304 (74 %)	**0.12**
	Yes	38 (22 %)	67 (29 %)	105 (26 %)	
Localization of the primary tumor	Colon	136 (78 %)	184 (78.5 %)	320 (78 %)	**0.79**
	Rectum	39 (22 %)	48 (20.5 %)	87 (21.5 %)	
	Unknown	0	2 (1 %)	2 (0.5 %)	
EGFR therapy	Yes	63 (36 %)	104 (44.5 %)	167 (41 %)	**0.11**
	No	112 (64 %)	130 (55.5 %)	242 (59 %)	
No. of treatment lines	1	32 (18 %)	19 (8 %)	51 (12.5 %)	**<0.0001**
	2	41 (23.5 %)	30 (13 %)	71 (17 %)	
	3 or more	102 (58.5 %)	185 (79 %)	287 (70.5 %)	
No. of metastatic sites	1	110 (63 %)	138 (59 %)	248 (60 %)	**0.48**
	>1	65 (38 %)	96 (41 %)	161 (40 %)	
CEA level	Median [min; max]	17 [0; 20,800]	18 [0; 14,660]	18 [0; 20,800]	**0.6**
	Mean (SD)	540 (2,405)	380 (1,440)	447 (1,961)	

*WHO* World Health Organization, *PS* performance status, *EGFR* epidermal growth factor receptor, *CEA* carcinoembryonic antigen

### Patients from Validation Cohort

Between January 1997 and December 2009, 328 mCRC patients were treated at the Besancon University Hospital. Of these, 177 patients (54 %) received bevacizumab during their chemotherapeutic treatment for metastatic disease. Patients and tumor characteristics are provided in Supplementary Table S1. As for the study cohort, there was no significant difference between the bevacizumab and chemotherapy-alone groups for the main available clinicobiological characteristics, except for anti-EGFR treatment, which was more frequently received by patients in the bevacizumab group (53 vs. 26 %, *P* *<* 0.0001). Median follow-up at the data cutoff point was 23 months in the bevacizumab group and 17 months in the chemotherapy-alone group.

### OS in Bevacizumab and Chemotherapy-alone Groups in the Study Cohort

**Fig. 1:**
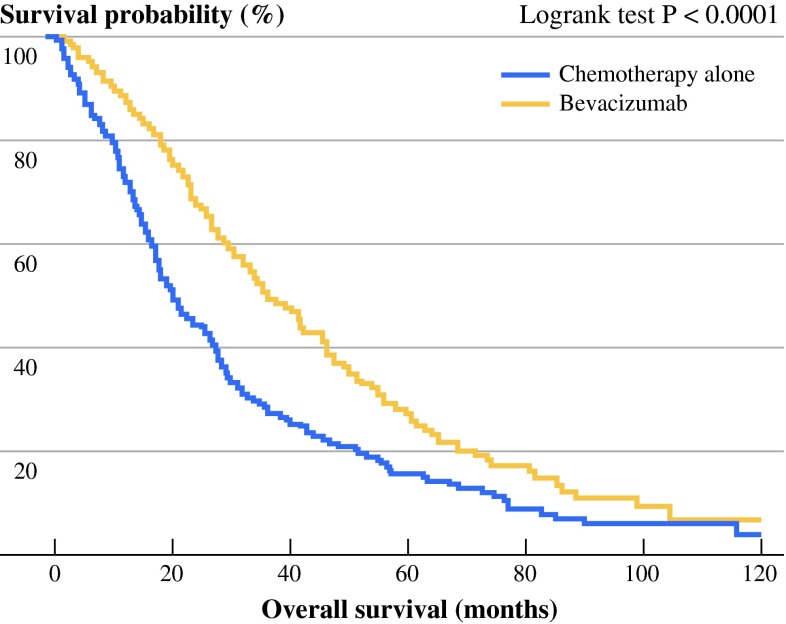
Kaplan–Meier *curve* for OS in the study cohort (*n* = 409) of mCRC patients, stratified according to treatment: chemotherapy with bevacizumab (bevacizumab group), or chemotherapy without bevacizumab (chemotherapy-alone group). *P* value was calculated by log-rank test

Proportions of patients who died were 88 % in the chemotherapy-alone group, and 67.5 % in the bevacizumab group (*P* < 0.0001). Patients receiving bevacizumab had a better outcome in term of OS compared to patients from the chemotherapy-alone group (log-rank test *P* < 0.0001) (Fig. 1). Median OS was 35.8 months with bevacizumab and 20.1 months without bevacizumab. Univariate analysis indicates that age, high CEA level, WHO performance status ≥2, B-Raf mutated tumor status, synchronous metastatic disease, absence of complete surgery of metastases, localization of primary tumor in colon, absence of anti-EGFR therapy, and more than 1 metastatic site were significantly associated with poorer OS (Table 2). Bevacizumab use (HR 0.61, 95 % CI 0.49–0.77, *P* < 0.0001) and primary tumor resection (HR 0.32, 95 % CI 0.24–0.43, *P* < 0.0001) were significantly associated with improved OS (Table 2).

**Table 2:** Univariate and multivariate analysis (Cox regression) for factors associated with overall survival

Characteristic	Variable	Univariate analysis			Multivariate analysis		
		HR	95 % CI	*P* value	HR	95 % CI	*P* value
Age^a^		1.0137	1.0004–1.0272	**0.04**	1.0044	[0.99; 1.02]	**0.54**
Sex	Male	1					
	Female	1.05	0.84–1.30	**0.67**			
CEA level^a^		1.001	1.0001–1.0002	**0.0001**	1.0001	[1.0000; 1.0002]	**0.0002**
WHO PS	0–1	1					
	≥2	2.75	1.97–3.85	**0.001**			
B-Raf status	Wild type	1					
	Mutated	2.29	1.19–4.40	**0.013**			
K-Ras status	Wild type	1					
	Mutated	1.42	0.98–2.03	**0.06**			
Evolution	Metachronous	1					
	Synchronous	1.35	1.08–1.7	**0.01**			
Primary tumor resection	No	1			1		
	Yes	0.32	0.24–0.43	**<0.0001**	0.36	[0.26; 0.49]	<**0.0001**
Complete surgery of metastases	Yes	1			1		
	No	2.56	2–3.45	**<0.0001**	0.48	[0.34; 0.67]	<**0.0001**
Localization of primary tumor	Rectum	1					
	Colon	1.33	1.01–1.75	**0.04**			
Anti-EGFR therapy	Yes	1			1		
	No	1.35	1.09–1.69	**0.008**	0.80	[0.61; 1.04]	**0.1**
No. of metastatic sites	1	1			1		
	>1	1.30	1.11–1.51	**0.012**	1.05	[0.88; 1.27]	**0.55**
Bevacizumab use	No	1			1		
	Yes	0.61	0.49–0.77	<**0.0001**	0.64	[0.50; 0.81]	**0.0007**

^a^Hazard ratio for continuous variable was calculated for 1 unit

*HR* hazard ratio, *CI* confidence interval, *CEA* carcinoembryonic antigen, *WHO* World Health Organization, *PS* performance status, *EGFR* epidermal growth factor receptor

By multivariate analysis, high CEA levels and absence of complete removal of metastases remained independently associated with poorer OS, bevacizumab use (HR 0.64, 95 % CI 0.50–0.81, *P* < 0.0007), and primary tumor resection (HR 0.36, 95 % CI 0.26–0.49, *P* < 0.0001) (Table 2).

### Effect of Primary Tumor Resection and Bevacizumab Use on OS in the Study Cohort (*n* = 409)

Because our initial hypothesis was that primary tumor resection could affect bevacizumab antitumor efficacy, we performed a subgroup analysis to assess the association of survival improvement with bevacizumab use both in patients who initially underwent primary tumor resection (*n* = 333) and in nonresected patients (*n* = 76). Both groups of patients with or without primary tumor resection were comparable for classical prognostic factors (Table 3). We only noted a significantly higher number of patients who received 3 or more treatment lines in the primary tumor resection group, which was probably linked to the longer survival in these patients and which is a classical problem of immortal time bias.^16^

**Table 3:** Patient and tumor characteristics (*n* = 409)

Characteristic	Variable	No. resection of primary tumor (*n* = 76)	Resection of primary tumor (*n* = 333)	Overall (*n* = 409)	*P* value, χ^2^ test
Age, (year)	Median [min; max]	65.1 [35; 86]	65.5 [24; 90]	65.5 [24; 90]	**0.98**
	Mean (SD)	66.2 (10.3)	64 (11.5)	65 (11)	
Sex	Male	37 (48.5 %)	183 (55 %)	220 (53.5 %)	**0.33**
	Female	39 (51.5 %)	150 (45 %)	189 (46.5 %)	
Death		65	251	312	**0.04**
WHO PS	0–1	48 (63 %)	194 (58 %)	242 (59 %)	**0.12**
	2–3	14 (18.5 %)	30 (9 %)	44 (11 %)	
	Unknown	14 (18.5 %)	109 (33 %)	123 (30 %)	
B-Raf status	Wild type	15 (20 %)	91 (27.5 %)	106 (26 %)	**0.19**
	Mutated	4 (5 %)	8 (2.5 %)	12 (3 %)	
	Unknown	57 (75 %)	234 (70 %)	291 (71 %)	
K-Ras status	Wild type	17 (22 %)	96 (29 %)	113 (27 %)	**0.95**
	Mutated	14 (18 %)	75 (22.5 %)	89 (22 %)	
	Unknown	45 (60 %)	162 (48.5 %)	207 (51 %)	
Evolution	Synchronous	76 (100 %)	179 (54 %)	254 (62 %)	<**0.0001**
	Metachronous	0 (0 %)	153 (46 %)	153 (38 %)	
Complete surgery of metastases	No	56 (73.5 %)	251 (75 %)	307 (74 %)	**0.87**
	Yes	20 (26.5 %)	82 (25 %)	102 (26 %)	
Localization of the primary tumor	Colon	59 (78 %)	262 (78.5 %)	320 (78 %)	**0.87**
	Rectum	16 (22 %)	71 (20.5 %)	87 (21.5 %)	
	Unknown	0	2 (1 %)	2 (0.5 %)	
EGFR therapy	Yes	30 (39.5 %)	137 (41 %)	167 (41 %)	**0.65**
	No	46 (40.5 %)	196 (59 %)	242 (59 %)	
Bevacizumab use	Yes	36 (47 %)	139 (42 %)	175 (43 %)	**0.54**
	No	40 (53 %)	194 (58 %)	234 (57 %)	
No. of treatment lines	1	15 (20 %)	36 (11 %)	51 (12.5 %)	**0.01**
	2	19 (25 %)	52 (16 %)	71 (17 %)	
	3 or more	42 (55 %)	245 (73 %)	287 (70.5 %)	
Metastases involving sites	1	40 (53 %)	208 (62 %)	248 (60 %)	**0.15**
	>1	36 (47 %)	125 (38 %)	161 (40 %)	
CEA level	Median [min; max]	141 [0; 14660]	14 [0; 20800]	18 [0; 20800]	**0.17**
	Mean (SD)	747 (2160)	370 (1902)	447 (1961)	

*WHO* World Health Organization, *PS* performance status, *EGFR* epidermal growth factor receptor, *CEA* carcinoembryonic antigen

**Fig. 2:**
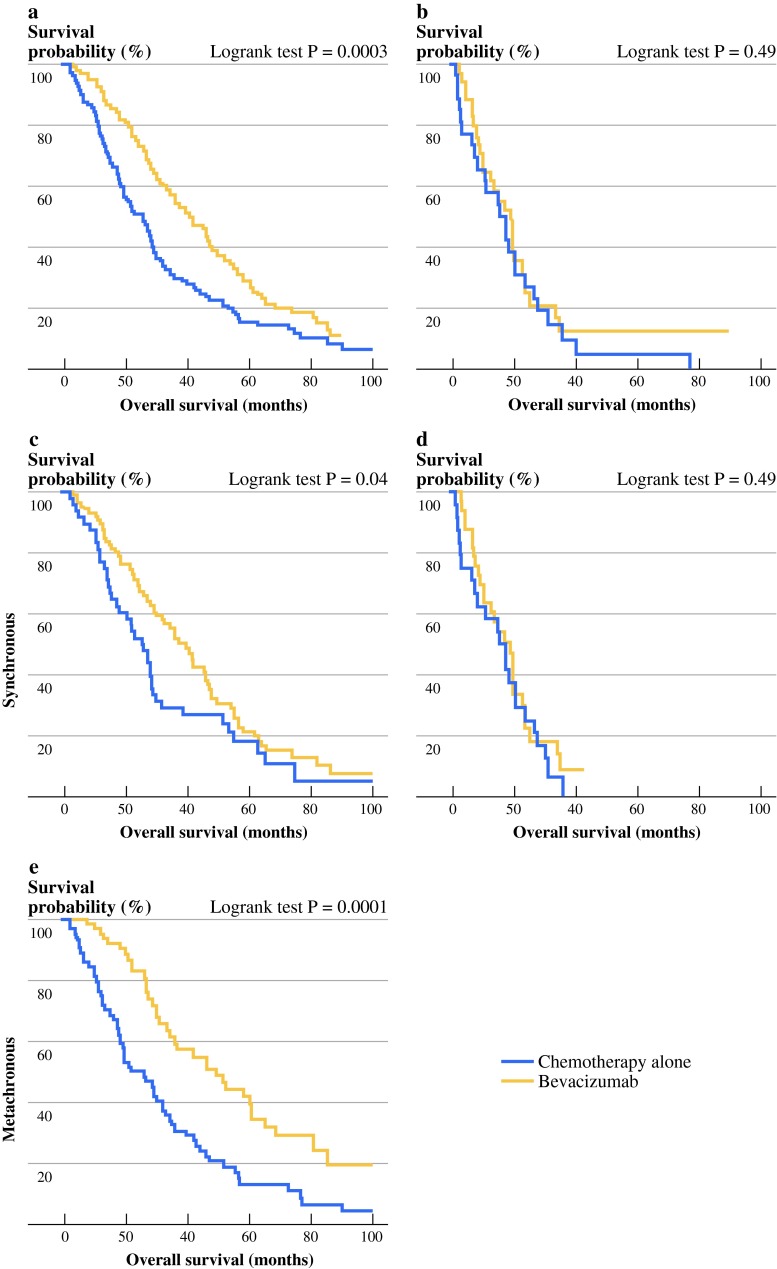
Kaplan–Meier *curves* for OS according to treatment: chemotherapy with bevacizumab (bevacizumab group), or chemotherapy without bevacizumab (chemotherapy-alone group), in patients who previously underwent primary tumor resection (*n* = 333) **a** and in patients who did not (*n* = 76) **b**. Kaplan–Meier curves for OS according to treatment: chemotherapy with bevacizumab (bevacizumab group), or chemotherapy without bevacizumab (chemotherapy-alone group), in patients with synchronous metastatic disease who previously underwent primary tumor resection (*n* = 153) **c** and in patients with synchronous metastatic disease and without primary tumor resection (*n* = 76) **d** and in patients with metachronous metastatic disease (*n* = 180) **e**. *P* values were calculated by log-rank test

Survival analysis revealed that the addition of bevacizumab failed to improve mCRC patient’s survival when primary tumor was present. In these patients, median OS was 18.5 months for the bevacizumab group and 17.1 months in the chemotherapy-alone group (HR 0.98, 95 % CI 0.60–1.61, log-rank test *P* = 0.6) (Fig. 2b). Nevertheless, as previously demonstrated, bevacizumab use significantly improved OS in patients who underwent primary tumor resection before beginning chemotherapy. In these patients with primary tumor resection, median OS was 41.9 months in the bevacizumab group and 25.3 months in the chemotherapy-alone group (HR 0.61, 95 % CI 0.47–0.79, log-rank test *P* = 0.0003) (Fig. 2a). By subgroup analysis and multivariate Cox model, we found that bevacizumab remained an independent prognostic factor of good outcome only in the group of patients with primary tumor resected (data not shown).

Interestingly, in patients who underwent primary tumor resection, we also observed that bevacizumab use was significantly associated with improved OS both in patients with metachronous metastases (HR 0.45, 95 % CI 0.30–0.68, *P* < 0.0001) (median OS 49.3 months when treated with bevacizumab vs. 25.6 months when treated with chemotherapy alone) (Fig. 2c) and in patients with synchronous metastases (HR 0.74, 95 % CI 0.50–0.98, *P* = 0.04) (median OS 41 months when treated with bevacizumab vs. 24.8 months when treated with chemotherapy alone) (Fig. 2d). Again, such significant differences in survival were not observed in nonresected patients (Fig. 2e). Interestingly, subgroup analysis emphasized that bevacizumab used as a first-line therapy or after first-line therapy improved survival of patients with primary tumor resection (*P* = 0.01 and *P* = 0.02, respectively) but not patients without primary tumor resection.

### Effect of Primary Tumor Resection and Bevacizumab Use on OS in the Validation Cohort (*n* = 328)

As in the study cohort, survival association of bevacizumab was assessed in patients who initially underwent primary tumor resection (*n* = 232) and in nonresected patients (*n* = 96). Both groups of patients with or without primary tumor resection were comparable for the available classical prognostic factors (Supplementary Table S2). As in the study cohort, bevacizumab improved OS in the whole population (median OS 23 months when treated with bevacizumab vs. 16 months when treated with chemotherapy alone) (HR 0.67, 95 % CI 0.52–0.85, *P* = 0.0008) (Supplementary Fig. S1A). The effect of bevacizumab on OS was significant only in patients who underwent primary tumor resection before beginning treatment for mCRC. In these patients with primary tumor resection, median OS was 27 months in the bevacizumab group and 16 months in the chemotherapy-alone group (HR 0.65, 95 % CI 0.49–0.88, log-rank test *P* = 0.0035) (Supplementary Fig. S1B). In contrast, bevacizumab use did not affect OS in nonresected patients. In these patients, median OS was 20 months in the bevacizumab group and 17 months in the chemotherapy-alone group (HR 0.73, 95 % CI 0.48–1.12, log-rank test *P* = 0.15) (Supplementary Fig. S1C).

## Discussion

This retrospective study demonstrated that the improved survival associated with the addition of adding bevacizumab to fluoropyrimidine-based chemotherapy appears to be dependent on the presence of the primary tumor in mCRC patients.

In mCRC patients, the role of resection of the primary tumor remains unclear. Case reports suggested that resection of the primary tumor could increase the growth rate of liver metastases on increased vascular density, proliferation rate, and metabolic growth rate.^17^–^19^ Taken together, these data suggest that the behavior of metastatic disease could be dependent on the primary tumor. Interestingly, several retrospective clinical studies have reported improved outcome of patients with mCRC who underwent primary tumor resection compared to nonresected patients.^20^–^22^ Unfortunately, the use of systemic therapy has not been recorded in some of these studies.

A first hypothesis to explain the detrimental effect of primary colorectal cancers on bevacizumab efficacy is the presence of metastatic dormancy imposed by the primary tumor. However, our data indicate that the presence of the primary tumor is correlated to a shorter OS, even in the absence of bevacizumab, ruling out the possibility that primary colon cancers reduce the kinetic of metastatic growth. This observation is supported by a retrospective analysis of two phase III clinical trials (CAIRO and CAIRO2). This study also underlined the favorable prognostic value of primary tumor resection in mCRC patients in terms of OS improvement.^20^

Preclinical studies raised caution on the antitumoral effects of antiangiogenic agents when primary tumor is in place. Indeed, in some animal models, angiogenesis inhibition can alter the natural history of tumors by increasing invasion and metastasis. In mCRC, the proangiogenic network in the tumor microenvironment could be different between the primary tumor and its related metastases. Indeed, it has been reported that VEGF expression is higher in colorectal cancer metastases than in the primary tumor.^23^ Moreover, colorectal cancer cell lines isolated from primary tumor were characterized by a higher expression of multiple proangiogenic factors, such as placental growth factor, thrombopoietin, and transforming growth factor β1, compared to tumor cell lines isolated from metastases.^14^ Taken together, these data are consistent with a more complex tumor microenvironment angiogenic network in the primary tumor than in the metastases. Thus, because metastases may be more dependent on VEGF-driven angiogenesis, targeting VEGF with bevacizumab may be more effective on metastases than on the primary tumor.

Moreover, in experimental tumor models, VEGF inhibition may also promote tumor invasion and could enhance metastasis.^24^ In these experiments, despite an initial antitumor benefit, antiangiogenic therapies may facilitate induction of invasive and metastatic tumor outgrowths. This in turn could limit the overall benefits in terms of OS. Ebos et al.^25^ observed in other animal models that although VEGFR inhibitor could delay the growth of orthotopically implanted tumors, such treatment also induced accelerated metastatic dissemination. Together, such data provide a biological rationale for the clinical results observed in our study, suggesting that systemic reactions to VEGF inhibition could facilitate tumor dissemination and may impede bevacizumab efficacy when primary tumor stays in place.

Limitations of our study include its two-center patient recruitment and its retrospective design. However, the use of two large independent cohorts, the homogeneity of results between both cohorts, and their separate analysis strongly confirm our original observation.

Moreover, the reason behind the decision of primary tumor resection had not been recorded, so we cannot exclude a confounding factor. In addition, the separation of patients into four groups reduces the number of patients per group and may impact on outcome. However, a comparison of patients who underwent resection of the primary tumor with unresected patients did not reveal in any differences in classical prognosis factors.

In conclusion, our results raised the possibility that bevacizumab is associated with improvement in OS only in patients with primary tumor resection, and thus our findings indicate a possible new predictive marker of bevacizumab efficacy. On the other hand, it seems that patients without primary tumor resection obtain no survival advantage from bevacizumab use. Such information is important and may reduce health care costs and toxic events in the subgroup of patients with primary tumor in place. Moreover, our results could explain, at least in part, conflicting results among clinical trials concerning bevacizumab efficacy in mCRC patients, underscoring the need to report the proportion of patients with primary tumor resection.

These data support the rationale to prospectively validate the influence of primary tumor resection on bevacizumab efficacy in synchronous stage IV colorectal cancer.

## Electronic Supplementary Material

Below is the link to the electronic supplementary material.

Supplementary material 1 (EPS 856 kb)

Supplementary material 2 (DOC 93 kb)

Supplementary material 3 (DOC 88 kb)

Supplementary material 4 (DOCX 10 kb)

Supplementary material 5 (EPS 1148 kb)

Supplementary material 6 (EPS 693 kb)
