# Supplementary material for: Bevacizumab Efficacy in Metastatic Colorectal Cancer is Dependent on Primary Tumor Resection
Source: Ann Surg Oncol. 2014 Jan 14;21(5):1632–40. doi: 10.1245/s10434-013-3463-y (PMC3975091; doi:10.1245/s10434-013-3463-y)
Supplement: Supplementary file 2 — Supplementary material 2 (DOC 93 kb) [file 10434_2013_3463_MOESM2_ESM.doc]

| **Supplementary Table 1 :** Patient and tumor characteristics validation cohort (n= 328): | | | | | |  |
| --- | --- | --- | --- | --- | --- | --- |
|  |  | **Chemotherapy alone** | **Bevacizumab** | **Overall** | **p-value** |  |
|  |  | **N=151** | **N=177** | **N=328** |  |  |
| **Age (year)** |  |  |  |  |  |  |
|  | median (min;max) | 67 [31;80] | 65 [35;80] | 66 [31;80] | **0.34** |  |
|  | mean(sd) | 65 (9) | 64.6 (9) | 64.8 (9) |  |  |
| **Sex** |  |  |  |  |  |  |
|  | male | 93 (62%) | 103 (58%) | 196 (59,8%) | **0.33** |  |
|  | female | 58 (38%) | 74 (42%) | 132 (40,2%) |  |  |
| **Death** |  |  |  |  |  |  |
|  |  | 138 (91%) | 129 (73%) | 267 | **<0.0001** |  |
| **WHO PS** |  |  |  |  |  |  |
|  | 0 | 9 (6%) | 71 (40%) | 80 (24%) | **0.002** |  |
|  | 1 | 6 (4%) | 33 (19%) | 39 (12%) |  |  |
|  | 2 | 7 (5%) | 5 (3%) | 12 (4%) |  |  |
|  | 3 | 1 (0.5%) | 2 (1%) | 3 (1%) |  |  |
|  | Unknown | 128 (84.5%) | 65 (37%) | 193 (59%) |  |  |
| **B-Raf Status** |  |  |  |  |  |  |
|  | Wild-Type | ND | ND | ND | **ND** |  |
|  | Mutated | ND | ND | ND |  |  |
|  | Unknown | ND | ND | ND |  |  |
| **K-Ras status** |  | |  |  |  |  |
|  | Wild-Type | 8 (5%) | 56 (32%) | 64 (20%) | **<0.0001** |  |
|  | Mutated | 5 (3%) | 39 (22%) | 44 (13%) |  |  |
|  | Unknown | 138 (92%) | 82 (46%) | 220 (67%) |  |  |
| **Evolution*** |  |  |  |  |  |  |
|  | Synchronous | 88 (59%) | 95 (59%) | 183 (59%) | **0.88** |  |
|  | Metachronous | 60 (41%) | 67 (41%) | 127 (41%) |  |  |
| **Primary tumor resection** |  |  |  |  |  |  |
|  | Yes | 100 (66%) | 132 (75%) | 232 (71%) | **0.09** |  |
|  | No | 51 (34%) | 45 (25%) | 96 (29%) |  |  |
| **Complete surgery of metastases** |  |  |  |  |  |  |
|  | No | ND | ND | ND | **ND** |  |
|  | Yes | ND | ND | ND |  |  |
| **Localization of the**  **primary tumor** |  |  |  |  |  |  |
|  | Colon | 99 (66%) | 122 (69%) | 221 (67%) | **0.52** |  |
|  | Rectum | 52 (34%) | 55 (31%) | 107 (33%) |  |  |
|  | Unknown | 0 | 0 | 0 |  |  |
| **Anti-EGFR therapy** |  |  |  |  |  |  |
|  | Yes | 39 (26%) | 93 (53%) | 132 (40%) | **<0.0001** |  |
|  | No | 112 (74%) | 84 (47%) | 196 (60%) |  |  |
| **Number of treatment lines** |  |  |  |  |  |  |
|  | 1 | 51 (34%) | 40 (23%) | 91 (28%) | **0.06** |  |
|  | 2 | 33 (22%) | 39 (22%) | 72 (22%) |  |  |
|  | 3 or more | 67 (44%) | 98 (55%) | 165 (50%) |  |  |
| **Number of metastatic sites** |  |  |  |  |  |  |
|  | 1 | 109 (72%) | 125 (71%) | 234 (71%) | **0.75** |  |
|  | >1 | 42 (28%) | 52 (29%) | 94 (29%) |  |  |
| **CEA level** |  |  |  |  |  |  |
|  | median (min;max) | ND | ND | ND | **ND** |  |
|  | mean(sd) | ND | ND | ND |  |  |

* Missing data (n = 18), ND : not determined
