# Supplementary material for: Bevacizumab Efficacy in Metastatic Colorectal Cancer is Dependent on Primary Tumor Resection
Source: Ann Surg Oncol. 2014 Jan 14;21(5):1632–40. doi: 10.1245/s10434-013-3463-y (PMC3975091; doi:10.1245/s10434-013-3463-y)
Supplement: Supplementary file 3 — Supplementary material 3 (DOC 88 kb) [file 10434_2013_3463_MOESM3_ESM.doc]

| **Supplementary Table 2 :** Patient and tumor characteristics validation cohort (n= 328): | | | | | | |  |
| --- | --- | --- | --- | --- | --- | --- | --- |
|  |  | | **No Resection of primary tumor** | **Resection of primary tumor** | **Overall** | **p-value** |  |
|  |  | | **N=96** | **N=232** | **N=328** |  |  |
| **Age (year)** |  | |  |  |  |  |  |
|  | median (min;max) | | 68 [45;80] | 65 [31;80] | 66 [31;80] | **0.07** |  |
|  | mean(sd) | | 66.4 (8.7) | 64.1 (9.7) | 64.8 (9.4) |  |  |
| **Sex** |  | |  |  |  |  |  |
|  | male | | 60 (62.5%) | 136 (59%) | 196 (60%) | **0.54** |  |
|  | female | | 36 (37.5%) | 96 (41%) | 132 (40%) |  |  |
| **Death** |  | |  |  |  |  |  |
|  |  | | 89 | 178 | 267 | **0.001** |  |
| **WHO PS** |  | |  |  |  |  |  |
|  | 0-1 | | 25 (26%) | 94 (41%) | 119 (36%) | **0.045** |  |
|  | 2-3 | | 7 (7%) | 9 (4%) | 16 (5%) |  |  |
|  | Unknown | | 64 (67%) | 129 (56%) | 193 (59%) |  |  |
| **B-Raf Status** |  | |  |  |  |  |  |
|  | Wild-Type | | ND | ND | ND | **ND** |  |
|  | Mutated | | ND | ND | ND |  |  |
|  | Unknown | | ND | ND | ND |  |  |
| **K-Ras status** | |  | |  |  |  |  |
|  | Wild-Type | | 14 (15%) | 50 (22%) | 64 (20%) | **0.02** |  |
|  | Mutated | | 7 (7%) | 37 (16%) | 44 (13%) |  |  |
|  | Unknown | | 75 (78%) | 145 (62%) | 220 (67%) |  |  |
| **Evolution** |  | |  |  |  |  |  |
|  | Synchronous | | 28 (31%) | 99 (45%) | 127 (41%) | **0.02** |  |
|  | Metachronous | | 63 (69%) | 183 (100%) | 183 (59%) |  |  |
| **Complete surgery of metastases** |  | |  |  |  |  |  |
|  | No | | ND | ND | ND | **ND** |  |
|  | Yes | | ND | ND | ND |  |  |
| **Localization of the**  **primary tumor** |  | |  |  |  |  |  |
|  | Colon | | 60 (63%) | 161 (69%) | 221 (67%) | **0.23** |  |
|  | Rectum | | 36 (37%) | 71 (31%) | 107 (33%) |  |  |
|  | Unknown | | 0 | 0 | 0 |  |  |
| **EGFR therapy** |  | |  |  |  |  |  |
|  | Yes | | 44 (46%) | 88 (38%) | 132 (40%) | **0.18** |  |
|  | No | | 52 (54%) | 144 (62%) | 196 (60%) |  |  |
| **Bevacizumab usage** |  | |  |  |  |  |  |
|  | Yes | | 45 (47%) | 132 (57%) | 177 (54%) | **0.11** |  |
|  | No | | 51 (53%) | 100 (43%) | 151 (46%) |  |  |
| **Number of treatment lines** |  | |  |  |  |  |  |
|  | 1 | | 21 (22%) | 70 (30%) | 91 (28%) | **0.26** |  |
|  | 2 | | 21 (22%) | 51 (22%) | 72 (22%) |  |  |
|  | 3 or more | | 54 (56%) | 111 (48%) | 165 (50%) |  |  |
| **Number of metastatic sites** |  | |  |  |  |  |  |
|  | 1 | | 62 (65%) | 172 (74%) | 234 (71%) | **0.08** |  |
|  | >1 | | 34 (35%) | 60 (26%) | 94 (29%) |  |  |
| **CEA level** |  | |  |  |  |  |  |
|  | median (min;max) | | ND | ND | ND | **ND** |  |
|  | mean(sd) | | ND | ND | ND |  |  |
|  |  | |  |  |  |  |  |

ND : not determined
