# Supplementary material for: Bevacizumab Efficacy in Metastatic Colorectal Cancer is Dependent on Primary Tumor Resection
Source: Ann Surg Oncol. 2014 Jan 14;21(5):1632–40. doi: 10.1245/s10434-013-3463-y (PMC3975091; doi:10.1245/s10434-013-3463-y)
Supplement: Supplementary file 4 — Supplementary material 4 (DOCX 10 kb) [file 10434_2013_3463_MOESM4_ESM.docx]

**Supplementary Figure 1:**

Kaplan-Meier curve for OS (**A**) in the whole validation cohort (N= 328) of mCRC patients, stratified according to treatment: chemotherapy + bevacizumab (bevacizumab group), or chemotherapy without bevacizumab (chemotherapy alone group), (**B**), in patients who previously underwent primary tumor resection (N=232), and (**C**) in patients without primary tumor resection (N=96). *P* value was calculated using the log-rank test.
